# Comparison of the cryo-tolerance of vitrified gorgonian oocytes

**DOI:** 10.1038/srep23290

**Published:** 2016-03-17

**Authors:** Sujune Tsai, Vivian Yang, Chiahsin Lin

**Affiliations:** 1 Department of Biotechnology, Mingdao University, 369 Wen-Hua Road, Peetow, ChangHua, 52345, Taiwan; 2 Department of Post Modern Agriculture, Mingdao University, 369 Wen-Hua Road, Peetow, Chang Hua, 52345, Taiwan; 3 Institute of Marine Biology, National Dong Hwa University, 2 Houwan Road, Checheng, Pingtung, 944, Taiwan; 4 National museum of Marine Biology & Aquarium, 2 Houwan Road, Checheng, Pingtung, 944, Taiwan

## Abstract

Coral reefs have been declining considerably in recent years because of changes to the environment and climate. The cryopreservation of coral gametes is an essential alternative method that yields immense success in preserving corals. This study focuses on developing vitrification techniques for *Junceella fragilis* and *Ellisella robusta* oocytes, and presents a comparison on the cryotolerance of their vitrified oocytes. The results revealed that these coral oocytes could be preserved for a longer period in equilibration solution 2 and vitrification solution (VS) 2 at 5 °C than at 26 °C. Oocyte viability decreased significantly when VS2 was used for >4 min at 26 °C compared with the control. Cryoprotectant tolerance was higher in *E. robusta* oocytes than in *J. fragilis* oocytes. However*, E. robusta* was determined to be more cryo-tolerant, with differences attributed to their habitats, thus making *E. robusta* is likely a superior candidate species for further study. The results of this study on the effects of coral cryopreservation provide a foundation for developing protocols further for the cryopreservation of the oocytes of gorgonian corals.

Corals are most essential to the marine ecosystem because they provide both food and shelter to several aquatic organisms. In recent years, corals have been disappearing rapidly, consequently affecting the entire ecosystem. Therefore, it is urgent that both research and preservation efforts be devoted to saving coral reefs for conserving marine life^1^,^2^. Several factors have contributed to the major depletion of corals in recent years, such as environmental and climate changes as well as diseases, and most prominently, human influence^3^,^4^. Overfishing has led to the loss of larger predators and an abundance of smaller organisms, resulting in competition and dominance over other species^5^. Furthermore, heavy pollution in oceans has drastically altered the marine habitat. In the event of natural disasters and damages, corals can regenerate; however, in recent years, this capability has decreased because of human disturbances^6^.

Cryopreservation involves preserving germ cells through freezing techniques for future breeding purposes. Because low temperatures are highly damaging to gametes, the primary concern in cryopreservation is in refining the freezing and thawing techniques for determining optimal conditions^7^,^8^. With current technologies, a variety of biological materials, including DNA, gametes, somatic cells, embryos, and tissues can be cryopreserved^9^,^10^. Although the field of invertebrate cryobiology lags behind that of the respective vertebrate field, freezing techniques have nevertheless been employed to cryopreserve eggs and embryos of a diverse array of invertebrate taxa, such as cnidarians (e.g. corals, *Junceella juncea*)^11^, mollusks (e.g. oysters, *Crassostrea virginica* and greenshell mussel, *Perna canaliculus*)^12^,^13^, polychaetes (e.g. *Nereis virens*)^14^, and echinoderms (e.g. sea urchins, *Cvechinus chloroticus*)^15^.

Vitrification is a technique used in cryopreservation, where cells are suspended in a mixture of cryoprotectant (CPA) or equilibration solution (ES) and vitrification solution (VS), and then submerged in liquid nitrogen. Under rapid freezing conditions, this mixture forms a glass-like structure with minimal ice crystal formation^16^. Vitrification is becoming a preferred technique for cryopreservation because it is efficient, economic, and minimises freezing injuries to the cells^11^. Vitrification differs from crystallisation because it does not involve molecule rearrangements in a crystal lattice structure. Regarding the former, it is clear from prior works that the oocytes of scleractinian corals are more sensitive to sub-zero temperatures than those of gorgonian corals, an observation that suggests that vitrification may be a preferred technique for the preservation of gorgonian oocytes^11^; briefly, this approach bypasses the hypothmic zone through direct contact of biological samples with liquid nitrogen. As such, the objective of the present study was to develop vitrification techniques for the ultimate cryopreservation of oocytes from gorgonian corals, *Ellisella robusta* and *Junceella fragilis*, with a focus on the cryotolerance of their vitrified oocytes.

## Results

### Vitrification properties of cryopreservant solutions

**Figure 1:**
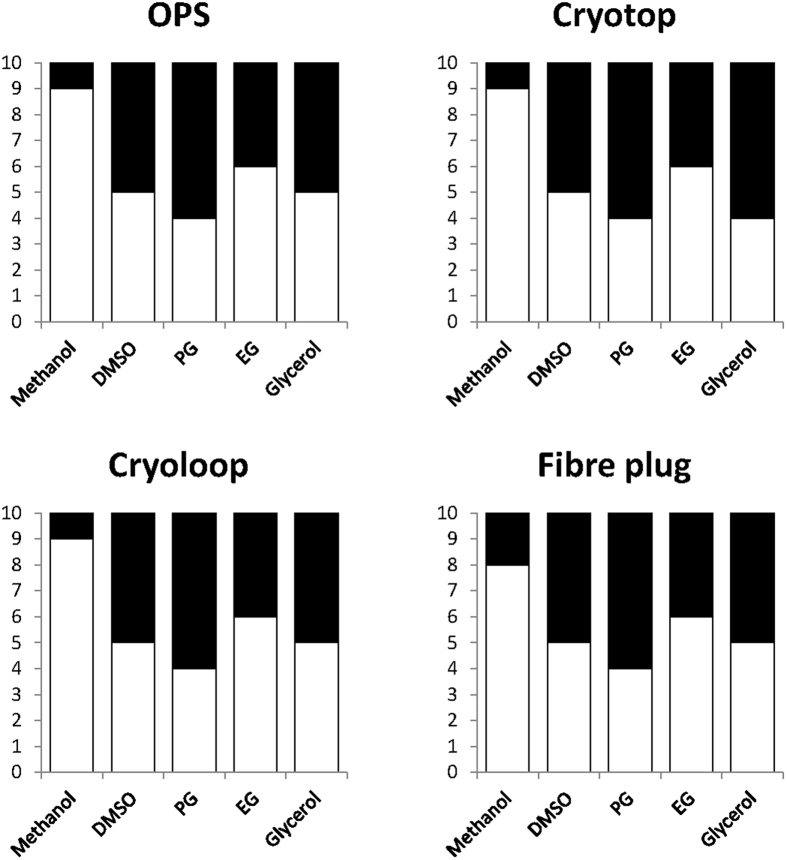
Minimum concentration (M) of CPAs with vitrification carriers. Black columns indicate vitrification when cooled in liquid nitrogen, whereas white columns represent devitrification.

Figure 1 shows the results of the effects of various cryopreservants (i.e. propylene glycol [PG], ethylene glycol [EG], methanol, glycerol, and dimethyl sulfoxide [DMSO]) along with different carriers (i.e. open pulled straw [OPS], cryotop, cyroloop, and fibre plug) used for the vitrification process. The order of minimum concentrations required for an effective outcome was as follows: PG < DMSO = glycerol < EG < methanol. PG was the solution that required the lowest concentration, and methanol was the solution that required the greatest concentration for vitrification. Fibre plug and cryotop were efficient carriers when used with methanol and glycerol, respectively, because vitrification was possible with a low concentration of these CPAs.

### Vitrification properties of VS mediums

**Figure 2:**
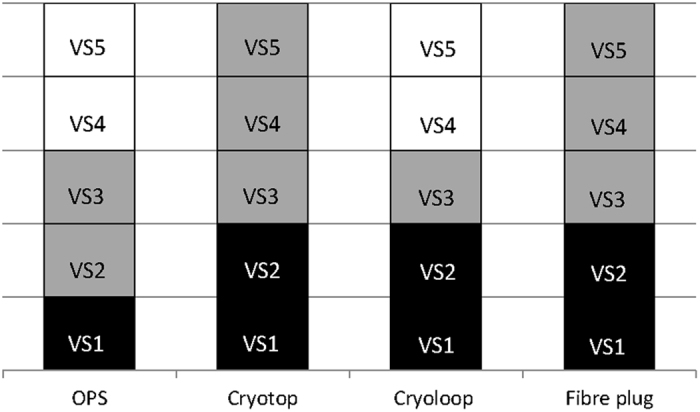
Examining PG-based VS with vitrification carriers. Black sections represent vitrification during freezing and thawing. Grey sections represent vitrification during freezing, but devitrification upon thawing. White sections represent crystallisation in liquid nitrogen.

The vitrifying properties of the PG-based VSs were examined, and the results are shown in Fig. 2. PG-based VS1 exhibited positive vitrification when used with all carriers. PG-based VS2 yielded successful vitrification with cryotop, cryoloop, and fibre plug. Although PG-based VS3 could vitrify when used with all carriers, it was devitrified upon thawing. By contrast, PG-based VS4 and VS5 used with OPS and Cryoloop did not exhibit vitrification under cooling and thawing conditions.

### ES1, ES2, VS2 on the viability of oocytes

**Figure 3:**
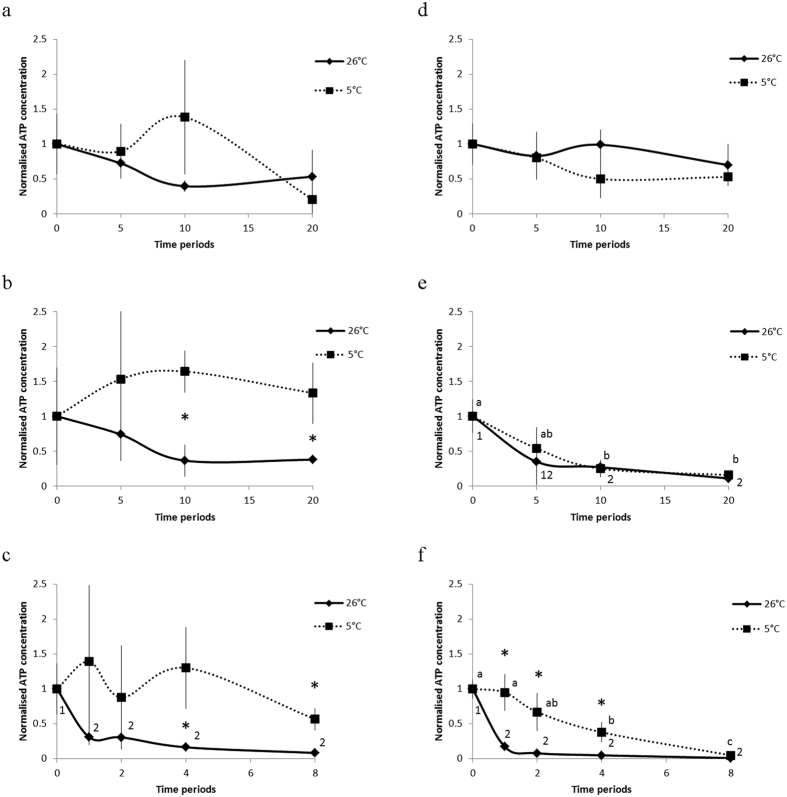
Effects of PG-based ES1, ES2, and VS2 on the *J. fragilis* and *E. robusta* oocytes at 5 °C and 26 °C for different time periods. Different letters and numbers represent statistical differences between exposure time points for oocytes treated with the same solutions (*P < 0.05 difference between temperatures).

The oocytes of *J. fragilis* and *E. robusta* submerged in ES1 did not exhibit any significant difference (*P* > 0.05) in viability at 26 °C and 5 °C, nor when these conditions were compared against those of the controls, and also in the comparisons for the final ATP counts at 20 min (Fig. 3a,d). When the *J. fragilis* oocytes were submerged in ES2, no difference was observed between their starting and final ATP counts at 26 °C and 5 °C. However, after 10 min, the viability of the *J. fragilis* oocytes at 5 °C was significantly higher than at 26 °C (*P* < 0.05), indicating that the oocytes endured better when vitrification was performed at 5 °C. This phenomenon was not observed in the *E. robusta* oocytes. The results revealed that the oocytes of *J. fragilis* and *E. robusta* could be preserved for longer in VS2 without a considerable decrease in viability at 5 °C than at 26 °C. The viability of the *J. fragilis* oocytes in VS2 did not vary significantly at 5 °C; however, the final viability decreased at 8 min. By contrast, the viability of the *E. robusta* oocytes decreased significantly after 4 min of incubation in VS2 at 5 °C (*P* < 0.05). The viability of the oocytes of both species started decreasing after incubation in VS2 for 1 min at 26 °C, indicating that at ≥1 min the oocytes were affected by VS2 treatment.

### Comparison of cryopreservant tolerance of the *J. fragilis* and *E. robusta* oocytes

**Figure 4:**
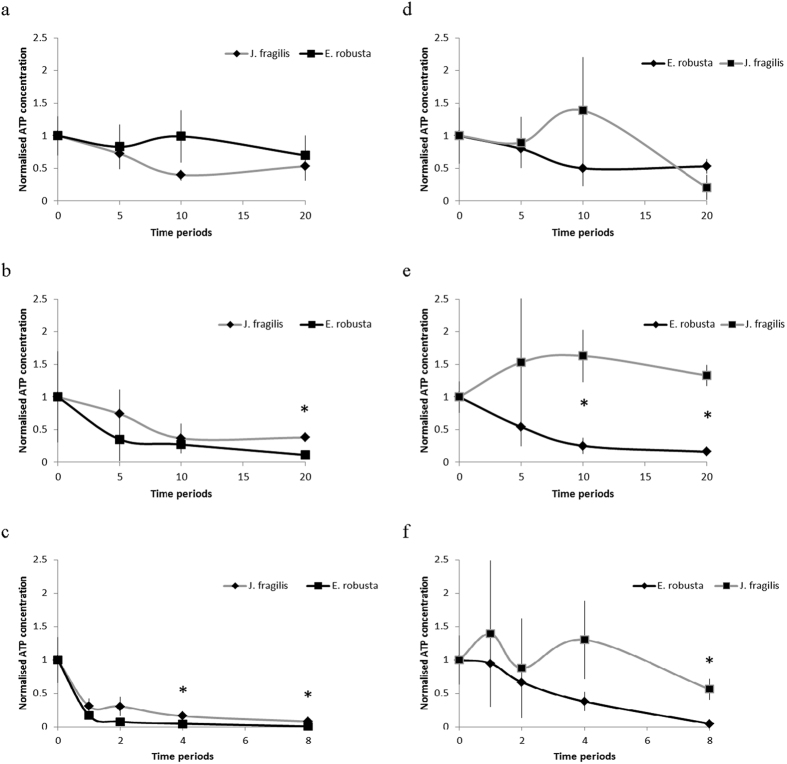
Comparison of the effects of ES1 (**a**,**d**), ES2 (**b**,**e**), and VS2 (**c**,**f**) at 26 °C and 5 °C at different time points for the *J. fragilis* and *E. robusta* oocytes. The black line represents *E. robusta* oocytes, and the grey line represents *J. fragilis* oocytes. * represents a significant difference between the 2 species.

Figure 4 shows a comparison between the oocytes of *J. fragilis* and *E. robusta* after being submerged in ES1 solution for 20 min at 26 °C and 5 °C. Because ES1 was used at lower concentrations compared with ES2 and VS2, the effect of ES1 on the oocytes was less obvious; consequently, determining a notable trend for the comparisons between viabilities of the *J. fragilis* and *E. robusta* oocytes under different conditions was difficult (Fig. 4a,d). The *J. fragilis* oocytes exhibited a high tolerance for ES2 at 26 °C and at 5 °C (Fig. 4b,e). The oocytes of *J. fragilis* and *E. robusta* initially exhibited similar responses to ES2; however, at 20 min, the viability of the *J. fragilis* oocytes was significantly higher than that of the *E. robusta* oocytes at both 26 °C and 5 °C (*P* < 0.05). The difference between the cryopreservant tolerance of the *J. fragilis and E. robusta* oocytes was more evident when VS2 was used. The ATP count of *J. fragilis* oocytes was significantly higher than that of the *E. robusta* oocytes at 2 min, 4 min, and 8 min (*P* < 0.05; Fig. 4c). Although initially no substantial difference was observed between the viabilities of the 2 types of oocytes, at 8 min the *J. fragilis* oocytes exhibited higher viability, indicating that they could tolerate the toxic effects of VS2 compared with the *E. robusta* oocytes. In summary, *J. fragilis* oocytes exhibited higher tolerance to VS than did the *E. robusta* oocytes (Fig. 4f).

### Vitrification

**Figure 5:**
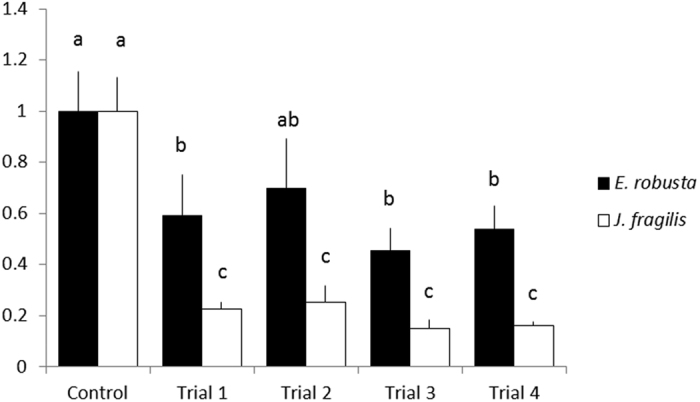
Normalised ATP Concentration in the *J. fragilis* and *E. robusta* oocytes after the vitrification assay using ES1, ES2, and VS2. Different letters represent statistical differences between exposure time points for oocytes treated with the same solutions (*P* < 0.05).

Among the 4 trials (Trials 1–4), none were found to yield a significant difference for oocyte viability between the *J. fragilis and E. robusta oocytes* (*P* < 0.05). Figure 5 shows a comparison between the 4 trials and controls. The viability of the *J. fragilis* oocytes was significantly lower than that of the control for all 4 treatments (*P* < 0.05), indicating that a stepwise vitrification process using ES1, ES2, and VS2 for the *J. fragilis* oocytes was not optimal. By contrast, the *E. robusta* oocytes responded better to the vitrification process; the results of Trial 1 and 2 revealed that the viability of the oocytes of *E. robusta* was lower than that of the controls; however, the difference was nonsignificant (*P* > 0.05). Trial 2 included the oocytes submerged in ES1 for the longest duration (15 min) before being transferred to ES2, indicating that treatment in ES1 for a long duration was required for protecting the oocytes before submerging them in a higher-concentration solution. The results of Trials 3 and 4 varied significantly compared with those of the controls (*P* < 0.05); however, the *E. robusta* oocytes endured better than the *J. fragilis* oocytes under the same conditions. These results revealed that the *J. fragilis* oocytes were more cryosensitive compared with the *E. robusta* oocytes.

## Discussion

Of the 4 types of carriers examined in this study, cryotop and fibre plug performed the best. The factors necessary for optimal vitrification are a fast cooling rate, a high-viscosity VS, and a small VS volume^17^. By reducing the VS volume and using carriers with a minimum capacity, heat conduction improves greatly for fast freezing^18^. Compared with OPS, cryotop and fibre plug use a substantially less amount of VS. Cryotop had the highest cooling rate compared with the other carriers used in this study. Fibre plug is typically used as a closed-carrier system; however, in this study, it was used as an open-carrier system. The cryotop carrier has been proven to be efficient for cattle, sheep, buffalo, and sensitive porcine and cattle oocytes^18^. The cryotop design allows several samples to be loaded simultaneously with minimal VS; thus, it was the preferred carrier for our study.

A crucial step in vitrification is in evaluating the cryoprotectant, a solution that shields cells from being exposed to extremely low temperatures. VS must be nontoxic to cells, and it must permeate through the cell membrane^16^. However, VS must be used at proper concentrations for complete vitrification; it is typically used at high concentrations because ice formation is reduced by increasing the amount of solutes^7^. In this study, VS2 was composed of 3.5M PG, 1.5M EG, and 2M methanol. The examination of the CPAs with the 4 carriers revealed that PG required the lowest concentration for completing vitrification, whereas methanol required the highest concentration compared with the other carriers. Previous studies have shown that methanol permeates throughout the cell membrane and protects coral oocytes, although it is used at high concentrations for complete vitrification^19^,^20^. As mentioned, a CPA with high viscosity is preferable for vitrification; thus, glycerol should have been a good candidate. However, in our previous experiments, we had compared glycerol- and PG-based VS under the same conditions as used in this study, and the results had shown that the PG-based VS yielded a suitable performance^11^. A similar study on zebrafish oocytes reported that DMSO was toxic, leading to various concerns, including protein denaturation and cytoskeletal reassembly^21^. However, another study on starfish oocytes reported that DMSO was an efficient CPA when used at low concentrations^22^.

VSs can also be toxic and detrimental to oocytes if they are directly submerged in VSs. It is essential to create ESs at low concentrations to ease interactions between the biological materials and the final VS in a stepwise manner. The viability of the oocytes submerged in ES1 did not exhibit significant differences at 5 °C and 26 °C; however, an observable difference was noted with ES2 and VS2. This result was noted for camel oocytes as well, which retained their metabolic levels at 4 °C than at 20 °C after 24 h^23^. At warm temperatures, oocytes metabolise, and their organelles develop. If an oocyte is subjected to experimentation at 26 °C under toxic conditions, it is still metabolically active, despite losing its major functions^23^. Storing oocytes at low temperatures slows their metabolic process, thus slowing the degenerative process^23^. CPA tolerance is conditional, and it depends strongly on the components used in a VS, the temperature of the treatment facility, the animal species, and the stage of oocyte development^7^. This explains the major difference between the viability of the *E. robusta* and *J. fragilis* oocytes after exposure to ES2 and VS2. Any combination of these factors could have affected our results.

One of the most frequent problems when performing a vitrification assay is in reducing osmotic shock, cell shrinkage, or expansion caused by the rapid loss or gain of water because of the differences in solute concentrations^24^. When an oocyte is submerged in the VS, the CPA essentially replaces the water present inside the oocyte^24^. If the solution is extremely hypertonic, the oocytes equilibrate through osmosis and undergo osmotic shock; however, this osmotic shock can be attenuated substantially with the slow introduction of the CPA^24^. Therefore, it was necessary to perform stepwise vitrification by using ES1 and ES2, 2 solutions at less toxic concentration levels, before adding VS2. This could also explain the high yield in viabilities obtained in Trials 1 and 2, which involved ES1 exposure for 15 min, compared with the rates obtained in Trials 3 and 4, which involved ES1 exposure for 10 min. Submerging cells in ES1 for a long period was beneficial because the oocytes had more time to equilibrate before being submerged in ES2.

In this study, the *E. robusta* oocytes were more cryotolerant than were the *J. fragilis* oocytes; however, the *E. robusta* oocytes were more CPA-sensitive. Our previous study reported that *J. juncea* oocytes have a higher lipid content compared with *J. fragilis* oocytes^25^. These differences were attributed to dissimilarities in their habitat, where *J. fragilis* is primarily found at depths of 3–5 m, whereas *E. robusta* is found at depths <20 m. Presumably, *E. robusta* has adapted to living under greater depths because its membrane has a high lipid composition, which allows for better membrane fluidity and protection from colder temperatures^25^,^26^. The lipid content of late-stage coral oocytes was also less than that of their early-stage counterparts; however, late-stage *E. robusta* oocytes had a higher lipid composition compared with late-stage *J. fragilis* oocytes. This can explain why the *E. robusta* oocytes were more cryotolerant than were the *J. fragilis* oocytes. A similar study was performed on starfish oocytes by using CPAs, DMSO, EG, and glycerol. First, CPA toxicity was examined^22^. In that study, the VS was composed of trehalose, DMSO, and artificial seawater in aluminum pans and cryostraws, and the optimal treatment led to a 34% survival rate^22^. Sugar, which is impermeable through the cell membrane, offsets the osmotic effect through a toxic VS, and many organisms naturally produce sugar when under stress. Furthermore, the surviving oocytes are fertilised and exhibit signs of normal development^5^. Instead of a stepwise addition of the VS, in the study on the cryopreservation of starfish oocytes, stepwise removal was performed during the thawing process. Although the methods differed, in our study, VS2 did not contain any sugar, which could be a potential area of improvement for future experiments.

Our study substantiated the difficulty in cryopreservation; no fixed method exists for optimal cryopreservation because multiple factors could affect cell survival. However, it was necessary to perform a series of trial experiments to identify the most efficient conditions. The results of our study revealed that the *J. fragilis* and *E. robusta* oocytes maintained viability better when treated at 5 °C than at 26 °C. After comparing the 2 species of gorgonian corals, we found *J. fragilis* to be more CPA-tolerant, whereas *E. robusta* was more cryotolerant, The *J. fragilis* and *E. robusta* colonies were typically found at 3–5, and <20 m depth, respectively. The collection depth difference between the former and latter species may have contributed to the variation in cryotolerance; specifically, *E. robusta* was determined to be more cryo-tolerant, and, as such, is a better candidate along with *J. juncea* for further studies. The results of this study on the effects of coral cryopreservation provide a foundation for developing protocols further for the cryopreservation of the oocytes of gorgonian corals.

## Methods

### Coral collection and oocyte isolation

*J. fragilis* and *E. robusta* were collected during reproduction seasons (June–September, 2011–2014) by divers off the coast of Checheng Township, Pingtung County, Taiwan (21°5′60″N, 120°5′60″E) and transported to Coral Husbandry Center, where they were bound, secured to a substrate, and placed in gravel bases of half-ton tanks with a water flow system at 25 °C. Using a knife, a thin tissue was cut from the surface of the corals, where the gametes were developed to determine whether they were viable for testing. The coral gametes were visible under a dissection microscope (SZ51; Olympus, Tokyo, Japan); sperm appeared smaller and transparent, whereas oocytes were larger and opaque. Polyps on the coral exterior indicate sites of coral germ cells. A thin layer was sliced from the coral surface and examined under the dissection microscope. With a pair of tweezers, the oocytes were prodded out of the strip and collected using a pipette. Oocytes >250 μm were used in this study. These corals are not regulated under Taiwanese law, and coral collection was approved by Kenting National Park.

### VSs and carriers

To determine the minimum concentration of each type of cryoprotectant for complete vitrification, 5 CPAs, namely methanol, DMSO, PG, EG, and glycerol at different concentrations (1–10M) were prepared using 0.2-μm filtered seawater and frozen with liquid nitrogen. All chemicals were purchased from Sigma (St. Louis, MO, USA). A solution was considered vitrified if it appeared transparent and glass-like, indicating no ice crystal formation; however, it was considered devitrified if it formed ice crystals and appeared opaque. Different vitrification carriers, namely OPS (IMV Technologies, Paris, France), cryotop (Kitazato, Tokyo, Japan), cryoloop (Hampton research, Riverside, CA, USA), and fibre plug (CryoLogic, Melbourne, VIC, Australia), were evaluated with the solutions to examine the vitrification properties of the CPAs. Each solution was examined 3 times using each carrier. OPS, cryotop, cryoloop, and fibre plug were loaded with a micropipette and then placed in liquid nitrogen. Based on these results, 5 VSs (VS1: 4M PG + 1M EG + 2M methanol; VS2: 3.5M PG + 1.5M EG + 2M methanol; VS3: 3M PG + 2M EG + 2M methanol; VS4: 2.5M PG + 2.5M EG + 2M methanol; VS5: 2M PG + 3M EG + 2M methanol) were prepared and examined with each of the 4 carriers for 3 times under the mentioned conditions.

### ESs and VS on oocytes

Preparing ESs at low concentrations is essential because they facilitate the interaction between oocytes and the final VS in a stepwise manner. Oocytes were submerged in ES1 (1M PG + 0.5M EG + 0.5M methanol), and were examined at 5-min intervals for 20 min. After each interval, 5 oocytes were drawn from the solution, and an ATP count was performed, indicating the viability of the oocytes. The experiments were performed both at room temperature (approximately 26 °C) and in an ice bath (approximately 5 °C) for 3 times. These conditions were repeated for evaluating ES2 (2M PG + 1M EG + 1M methanol) and VS2 (3.5M PG + 1.5M EG + 2M methanol), except that the intervals for the VS2 experiment were at 1 min, 2 min, 4 min, and 8 min.

### Vitrification using ES1, ES2, and VS2

The effect of stepwise vitrification on the oocytes was determined using ES1, ES2, and VS2 at 5 °C by using the cryotop carrier. Fifteen oocytes were submerged in ES1 and then transferred to ES2 for various time intervals in each trial. During Trial 1, the oocytes were in ES1 and ES2 for 15 min and 10 min, respectively; 15 min and 5 min in ES1 and ES2, respectively, during Trial 2; 10 min in ES1 and ES2 each during Trial 3; and 10 min and 5 min in ES1 and ES2, respectively, in Trial 4. Finally, the oocytes were submerged in VS2 for 2 min. The oocytes were then placed on the cryotop carrier by using a pipette suction, and the carrier was submerged in liquid nitrogen for at least 10 min. After vitrification, the oocytes were immediately transferred back into VS2 at 5 °C for thawing and for preventing ice-crystal formation, and an ATP assay was performed to confirm oocyte viability. Each trial experiment was performed in triplicate.

### Viability assay

An ATP Assay was performed for determining the viability of the coral oocytes. The productivity of the mitochondria, as indicated by the number of ATPs, indicated the degree of harm for the oocytes in a treatment. Coral oocytes were placed in a test tube, and seawater was drawn using a pipette. Oocyte viability was assessed through a luminescence assay (ApoSENSOR Cell Viability Assay Kit, BioVision, Cambridge BioScience, UK). In brief, a nucleotide-releasing buffer (100 μL) was added to the test tube, and after 3–5 min, an ATP-monitoring enzyme (5 μL) was added. After approximately 30 s to 1 min, the test tube was placed into a luminometer (Lumat LB 9507, Berthold Technologies, Germany), and readings were taken^27^.

### Statistics

The normal distribution of the data was confirmed by performing a one-sample Kolmogorov–Smirnov test. Two-way analysis of variance was then performed to determine significant differences between the groups. Variance homogeneity was confirmed by conducting the Levene test (*P* > 0.05). All *P* values reported in the results section correspond to Tukey’s post-hoc differences between individual means; such tests were conducted only when a significant treatment effect was detected in the overall ANOVA model. The data are presented in the tables and figures as the mean ± SEM, and *P* values < 0.05 were considered statistically significant. All statistical data were generated using SPSS (version 17, IBM, Armonk, NY, USA).

## Additional Information

**How to cite this article**: Tsai, S. *et al.* Comparison of the cryo-tolerance of vitrified gorgonian oocytes. *Sci. Rep.* **6**, 23290; doi: 10.1038/srep23290 (2016).
